# Parents’ Agendas in Paediatric Clinical Trial Recruitment Are Different from Researchers’ and Often Remain Unvoiced: A Qualitative Study

**DOI:** 10.1371/journal.pone.0067352

**Published:** 2013-07-03

**Authors:** Kerry Woolfall, Valerie Shilling, Helen Hickey, Rosalind L. Smyth, Emma Sowden, Paula R. Williamson, Bridget Young

**Affiliations:** 1 Department of Psychological Sciences, University of Liverpool, Liverpool, United Kingdom; 2 Child Health Group, Exeter Medical School, Exeter, United Kingdom; 3 Medicines for Children Research Network Clinical Trials Unit, Alder Hey Children’s National Health Service (NHS) Foundation Trust, Liverpool, United Kingdom; 4 Institute of Child Health, University College London, London, United Kingdom; 5 Department of Biostatistics, University of Liverpool, Liverpool, United Kingdom; IUMSP, University Hospital Lausanne, Switzerland

## Abstract

Ensuring parents make an informed decision about their child’s participation in a clinical trial is a challenge for practitioners as a parent’s comprehension of a trial may differ from that intended by the practitioners responsible for recruitment. We explored what issues parents consider important when making a decision about participation in a paediatric clinical trial and their comprehension of these issues to inform future recruitment practice. This qualitative interview and observational study examined recruitment in four placebo-controlled, double-blind randomised clinical trials of medicines for children. Audio-recorded trial recruitment discussions between practitioners and parents (N = 41) were matched with semi-structured interviews with parents (N = 41). When making a decision about trial entry parents considered clinical benefit, child safety, practicalities of participation, research for the common good, access to medication and randomisation. Within these prioritised issues parents had specific misunderstandings, which had the potential to influence their decisions. While parents had many questions and concerns about trial participation which influenced their decision-making, they rarely voiced these during discussions about the trials with practitioners. Those involved in the recruitment of children to clinical trials need to be aware of parents’ priorities and the sorts of misunderstandings that can arise with parents. Providing trial information that is tailored to what parents consider important in making a decision about a clinical trial may improve recruitment practice and ultimately benefit evidence-based paediatric medicine.

## Introduction

The recognition that clinical trials are essential in developing safe and effective treatments for children has led to international policy changes to promote clinical research in paediatrics [1]–[3]. Despite such developments, there is on-going debate regarding the conduct of recruitment and consent methods for paediatric clinical trials at an international level [4]–[9]. Although parental consent is a legal and ethical requirement for children to be entered into a trial, a parent’s comprehension of their child’s involvement in the clinical trial may differ from that intended by the practitioners responsible for recruitment [7], [10]. Moreover, parents may sign consent forms and consider themselves informed without an adequate understanding of what the study entails or how involvement will impact upon family life [7], [10]. Ensuring parents make an informed decision about their child’s participation in a trial is a challenge for practitioners [11]–[13].

Investigations of parents’ interpretations of trials frequently identify ‘misunderstandings’, which, it is anticipated, can be remedied by improvements to written information provision [14], [15], sufficient time for participants to consider the information [16], and education for them and trial recruiters [7], [15], [17]. Parental misunderstandings most commonly arise in relation to the purpose of the trial, randomisation, eligibility and lack of equipoise [12], [18]–[21]. However, even when interventions are put in place to address misunderstandings, people will still interpret information in complex and unexpected ways, informed by their own priorities, belief systems, and trust in the practitioner. This raises questions regarding how far people’s interpretations can be altered to match those conventionally expected by the research community [11]. Masty and Fisher describe a goodness-of-fit approach, whereby researchers design consent procedures in ways that are tailored to participants’ priorities and well-being. In such an approach, a recruiter would focus on the issues that are of concern to potential participants, as well as aiming to help potential participants achieve the understanding of a trial that is conventionally expected by the research community [22].

Such a tailored or parent-centred approach may help to address the difficulties parents have in understanding trials, yet there is little evidence on what information parents consider important when they are approached about a trial and how well they understand this information. This study aimed to address this gap in the evidence-base by exploring what issues parents consider to be important in making a decision about their child’s participation in a clinical trial and parents’ comprehension of these prioritised elements. For example, randomisation is commonly misunderstood [23]. However, do parents consider having an understanding of randomisation to be important and would a fuller understanding assist their decision making? Giving more attention to information that parents deem important may assist practitioners’ recruiting to paediatric clinical trials by focussing on information that parents are most interested in, and which they will therefore be motivated to engage with.

We chose a qualitative approach in order to explore parental perspectives and decision-making during clinical trial recruitment. Importantly, we designed our qualitative study to compare what was said during trial recruitment discussions (which routinely take place before practitioners seek parental consent for a trial) with the interpretations that parents took away from these discussions. We therefore collected recordings of parent-practitioner clinical trial discussions as well as parent-researcher interviews.

## Methods

### Ethics Statement

A UK National Health Service ethics committee gave approval for the study (Northwest 5 Research Ethics Committee: 07/MRE08/6). Signed informed consent was obtained from all participants.

### Study Design

Our qualitative interview and observational study (called RECRUIT) ran alongside four diverse placebo-controlled, double-blind randomised clinical trials of medicines for children. Data triangulation based on comparisons of transcribed audio recordings of i) parent-practitioner recruitment discussions and ii) interviews with parents was used to examine recruitment processes with the aim of identifying strategies to improve clinical trial recruitment [24]. We selected trials to represent different conditions, disease status, design and recruitment procedures to maximise the transferability of findings and thereby inform strategies for optimising recruitment to children’s clinical trials [25], [26]. The trials comprised: MASCOT (Management of asthma in school children on therapy); MENDS (Use of melatonin in children with neuro-developmental disorders and impaired sleep); POP (prevention and treatment of steriod-induced osteopaenia in children and adolescents with rheumatic diseases); and TIPIT (Randomised controlled trial of thyroxine in pre-term infants under 28 weeks’ gestation). For logistical reasons, sites for inclusion in RECRUIT were generally selected from Northwest England. Recruitment approach and timing varied in all four trials. For example, TIPIT was initially introduced to parents by a practitioner, usually on the neonatal unit or at the mother’s bedside by a research nurse. In MASCOT, the initial approach was usually via letter from the GP or by a doctor when the child was attending a secondary care centre. Interested families received Participant Information Leaflets (PILs) and a telephone call from a research nurse before attending an appointment specifically arranged to discuss trial entry. Sociodemographic information was collected through the use of parents’ postcodes to calculate Index of Multiple Deprivation Scores (IMD) which indicate and rank levels of social deprivation in small geographical areas across the UK [27].

In sampling participants we used a mix of consecutive and purposive sampling. We used consecutive sampling initially to minimize potential gatekeeper selection bias, whereby practitioners may have selected families to avoid approaching based upon anticipated communication difficulties [28]. As the study progressed we increasingly used purposive sampling with the aim of ensuring that parents from each of the trials were represented and that those who declined, withdrew or who were ineligible for the trials were included, in addition to those who remained in the trial. A breakdown of sample trial trajectories are presented in Table 1. All families who had participated in audio recorded parent-practitioner trial discussions were invited to take part in interviews (except where a child had died or we could not contact the family) to help ensure that the sample variation represented in the trial discussion data was also reflected in the interview data. Importantly, this allowed us to link and compare data from both sources (i.e. patient-practitioner trial discussions and interviews) within cases and thereby, for example, to explore how a parent’s understanding of a trial might be associated with the way that the trial was explained during the discussion with a practitioner [29].

**Table 1:** Demographic and trial participation trajectory of parents.

	MASCOT	MENDS	POP	TIPIT
N (% of 41)	6 (14.6)	15 (36.6)	8 (19.5)	12 (29.3)
N Randomised	**4**	**9**	**8**	**11**
Decline trial	**0**	**1**	**0**	**1**
Ineligible for trial at consent	**1**	**1**	**0**	**0**
Ineligible for trial after run in	**1**	**3**	**N/A**	**N/A**
Withdrawn from trial	**0**	**1**	**0**	**0**

(N = 41 families).

### Research Team and Data Collection

Interviews were conversational, yet structured around a topic guide that covered areas which our review of literature and steering group members had indicated were pertinent. The topic guide was refined over the study course. Interviews were participant led to ensure that the content reflected their own priorities and the researchers aimed to explore participants’ perspectives in a way that avoided influencing, ‘testing’ or altering their beliefs about the trials. Respondent validation was used whereby previously unanticipated topics were added and discussed with participants as interviewing and analysis progressed [30]. Researcher notes were used to assist this process [29]. Topics included: the experience of trial recruitment from the perspective of parents; how they felt about the trial discussions; written and verbal information exchanged; whether any of this information was unclear; and whether anything might have been handled differently. See Shilling and colleagues 2011 (Text S2) [25]. Practitioners briefly explained RECRUIT to families who were eligible for each of the four trials before seeking verbal consent from parents to record the trial discussions and provide their contact details to the RECRUIT team. Semi-structured interviews with families were conducted by experienced interviewers VS and ES, who were psychologists with interests in clinical communication and clinical trial recruitment. Neither had any prior relationships with participants and no persons other than family members were present during the interviews. RECRUIT interviewers explained the study in more detail, including that its purpose was to identify ways to enhance the process of recruitment to children’s clinical trials. They also explained their independence from the trial and steps to ensure the confidentiality of the data before obtaining written consent from families who wished to participate. Practitioners securely sent audio recordings of the trial discussions to the RECRUIT team only after signed consent had been obtained from participants for the recordings to be released to the RECRUIT team. Audio recordings of trial discussions from parents who did not provide written consent were destroyed. Field notes were made by the researcher after each interview to assist analysis and interpretation. Parent-practitioner trial recruitment discussions and parent-researcher semi-structured interviews were digitally audio-recorded, transcribed verbatim, and checked and anonymised by VS and ES. We (KW, VS and BY) analysed transcripts of the parent-practitioner trial recruitment discussions alongside transcripts of semi-structured interviews.

### Analysis

Analysis was broadly interpretive and iterative, referring back and forth between the developing analysis and new data for evidence of families’ priorities, experiences and accounts of understanding when approached about a trial [31], [32]. Themes were therefore inductively derived from the data. Whilst analysis was informed by the constant comparison approach of grounded theory, the focus was modified to fit with the criterion of catalytic validity, whereby findings should be relevant to future research and practice [31], [33]. KW led the analysis and development of coding framework with assistance from VS and BY to enable investigator triangulation, and using QSR NVivo 9 software to assist in the organisation and indexing of coding and transcripts. KW read trial discussion and interview transcripts several times to compare within and between transcripts [31], [32]. Data triangulation was part of this process whereby she also compared interview data with trial discussion data. In particular, KW identified the issues that parents viewed as important when making a decision about a trial by systematically searching trial discussion and interview data for the questions asked by parents, the concerns that they voiced regarding their child’s participation in a trial and by examining parents’ interview accounts of their decision making process. Potential misunderstandings were identified by KW and VS by searching parents’ interview transcripts for descriptions or explanations that were not consistent with the trial rationale or methodology. We then cross-referenced these with transcripts from recorded trial discussions to examine whether the source of the misunderstanding could be potentially linked to the trial discussion. Potential important issues (which we refer to as ‘agenda items’) and misunderstandings were linked together by comparing and contrasting themes identified under each heading. The developing analysis and coding framework was discussed in detail with BY, who also read several of the transcripts. Detailed reports of the findings were reviewed and commented upon by all authors to help ‘test’ the analysis and ensure its rigour [29]. The quality and validity of developing analysis was examined by exploring the connection between emerging findings and the wider literature [24], [30]. Brief data extracts are provided in the main text of the results section and in illustrative tables to evidence our interpretations of participant accounts. Counts for each identified theme are presented in Tables 2 and 3 to make explicit the basis for conclusions drawn [34]. Data saturation was achieved with regard to parental agendas (Table 2). However, in terms of parental comprehension and the different types of misunderstanding that may arise linked to agenda items (Table 3), our data were more limited and we do not think saturation was achieved for this particular sub-category [35].

**Table 2:** Parents’ agenda items identified in trial recruitment discussions and interview analysis.

Agenda item	Number of parents referring to agenda item during interviews	Number of parents referring to agenda item in trial recruitment discussions
Clinical benefit	(16)	(3)
Safety	(15)	(5)
Practicalities of participation	(12)	(4)
Research for the common good	(12)	(0)
Access to medication	(9)	(4)
Randomisation	(7)	(1)
Contraindication	(6)	(1)
Showing gratitude to practitioner	(6)	(0)
Practitioner opinion	(5)	(0)
Child’s wishes	(4)	(0)
Trial purpose	(0)	(3)

(N = 41 families).

**Table 3:** Parent misunderstandings linked to important issues as identified in the interviews.

Misunderstandings (number of parents)	Example quotations	Context of misunderstanding shown in quotation
**Believed clinical benefit (8)**	*“I've been looking for something to help with her sleep problem for a long, long time.”* (Female 24)	Clinical benefit could not be guaranteed by participation in the trial.
**Access to medication (8)**	*“I’ve got sort of a 50/50 chance of either she gets the drug or she gets the placebo. But she wouldn’t be getting it otherwise”.* (Female 24)	Medication was available outside the particular trial.
**Randomisation (3)**	*“I wonder who does actually makes the decision, who goes on what and who doesn’t”.* (Female 1)	Computer randomisation. No person involved in making a decision.
**Practical implications of trial procedures (3)**	*“[Doctor] told me, I think, they’re going to put like kind of a small tube inside him…I just didn’t like the idea from the beginning so I didn’t give it more attention”.* (Female 34)	The trial medication was to be administered by a catheter already being used to administer drugs to the child.

(N = 41 families).

## Results

### Participants

A total of 95 families were approached by practitioners to participate in RECRUIT (65 approached for recorded discussion, 30 for interview without recorded discussion). Sixty families participated in RECRUIT (41 with recorded trial discussions, 19 without). Five families with recorded trial discussions were not approached for interview due to bereavement or contact difficulties (e.g. as they had been transferred to another hospital or did not respond to invitations). A further 30 families declined the study, either by direct refusal or by not arranging or cancelling appointments and not responding to further contacts from the research team. Families were not asked for their reasons for declining RECRUIT.

Of the total RECRUIT sample, 41 families had both recorded trial discussion and interview data, which were matched for analysis in this paper. Eleven practitioners were involved in the audio recorded trial discussions; of these 2 were research nurses and 9 were doctors. Table 1 indicates the numbers of families participating by trial and trajectory. The process of recruitment to clinical trials often involves several discussions between practitioners and potential participants. For 18 of the families (44%) in our study, the initial trial discussion that a family had with a practitioner was recorded. These were face to face discussions in which trial information was provided to families. The remaining 23 (56%) families had previously been given brief details of the trial by a practitioner, either over the telephone (19, 83%), or in person at a previous hospital visit (4, 17%). Recordings for these families relate to a subsequent face to face discussion with a practitioner in which information about the trial was presented. The initial trial discussions for these families were not recorded. Across all data included in the current analyses, there were eight instances where both parents were present for trial discussions (19.5%) and two instances where fathers were present at semi-structured interviews as well as mothers (5%). Only one member of the research team was present during the semi-structured interviews with parents; in a few instances, where it was unavoidable, a child (trial candidate or younger sibling) was present during the interviews. Interviews took place in the family home (n = 32, 78%), hospital site (n = 7, 17%) or via telephone (n = 2, 5%), occurred a mean of 42 days after the recorded trial discussion (range 14–126) and lasted approximately 45 to 60 minutes. As shown in Table 1, of the 41 families, 33 were randomised, though 1 later withdrew from the trial. Four consented but were ineligible after run in, 2 were ineligible for the trial at consent and 2 declined the trial. Interviews took place between March 2008 and January 2010.

The range of Index of Multiple Deprivation ranks (2007) in this sample was 1–30,591 with median rank 3560 and interquartile range 1366–10705 (where 1 is the highest level of deprivation). This excludes three families from Northern Ireland where deprivation scores are not directly comparable. Twenty four of the 38 (63%) families were in the lowest quintile (highest deprivation); only 3 were in the highest.

### Parents’ Agenda Items

We labelled the issues or topics that parents seemed to regard as important in making a decision about their child’s participation in a clinical trial as ‘agenda items’. Parents had many questions and concerns about trial participation that they raised spontaneously in their interviews when the researcher asked them to describe the trial consent procedure or design. As the following quote illustrates, parents placed importance upon issues such as child safety, which influenced their decisions about trial participation: *“If it involved* [child name] *taking part and it didn’t hurt them then yeah, by all means, go for it, do it, but if it hurts* [child name] *then I’d disagree with it completely*” (Male 2). Agenda items were evident in parents’ descriptions of decision making for all four trials.

As Table 2 shows the most common agenda items raised were a desire for their child to benefit from participation in the trial (clinical benefit): *“Yeah he can…. yeah take part yeah because I want…. I basically want him to like sleep, you know, have a good night's sleep”.* (Female 6, trial discussion), and concerns about whether their child would be harmed by participation (safety) *“So he wouldn’t become addicted to it?”* (Female 4, trial discussion). The practicalities of participation concerned parents, such as difficulties with children not wanting to take medicine: *“It’s really hard getting medication down, really hard”.* (Female 1, trial discussion). Parents described how research to benefit children in the future (research for the common good) had influenced their decision to participate: *“So as long as it can help in the future, other babies, then I’m really up for it”.* (Female 31, interview). Gaining access to medication for their child was also discussed: *“I’d heard about <name of trial drug> and I’d read a few things on the internet, because of <child’s name> sleeping, and I just thought, right I’m going to ask if he can have it”* (Female 8, interview). Randomisation was an issue for some parents, as indicated by their comments that the process could mean that their child would receive a placebo rather than the trial drug: *“We had to weigh it up against the fact that…. it could be a placebo anyway, it might not be the <trial medication>”.* (Female 24, interview).

Parents also questioned whether the trial medication could be taken alongside their child’s medication regime (contraindication) *“The questions that I've got to ask are just related to <child’s name> epilepsy really and just because we've had no seizure since his surgery in January…I think that's our only sort of concern…how it's gonna maybe interfere, with <non trial medication>”* (Female 6, trial discussion). For some parents showing gratitude to a practitioner was important: *“I just thought after everything they’ve done for us we can’t not, you know, give something back to them”* (Female 8, interview), whilst others pointed to a sense that they might be influenced by a practitioner’s opinion on whether their child may benefit from entering the trial: *“If a doctor did turn around and say to me…‘This is what I think you’d benefit from, this is what I think …’, you know, ‘… the child would benefit from and I think it’s a very good idea’ then the doctor could probably make my mind up for me actually”* (Female 1, interview). To a lesser extent parents described how their child’s wishes were important and how they had involved them in the decision making process: *“I always think it's best to be upfront with your kids, no matter what, to a level of their understanding…I mean, at nine, he's old…he is old enough to say, well, you know, I don't really fancy it”* (Female 18, interview). Finally, the purpose of the trial was questioned by some *“And what are you looking for in that?”* (Male 4, trial discussion). All parents had at least one agenda item, whilst some had multiple agenda items that informed their decision making, *“You just work out what […] you know, what happens? Will she be able to cope with it? Will it be beneficial to her or to others? And then, okay, yes, we'll do it or not”* (Male 14).

As shown by the differences in frequencies of parents referring to themes displayed in Table 2, agenda items were mainly discussed during the parent-researcher interviews, rather than in recorded trial discussions. For example, during interviews 12 parents discussed issues related to the practicalities of trial participation, such as administering medicine to children. This agenda item was only raised by four parents during trial discussions.

### Parents’ Misunderstandings Linked to Agenda Items

Parents often spoke of having a sense of confusion or poor recollection about the trial, which they linked to their emotional situation or being overloaded with information at the time of recruitment, but without specifying the particular issues they misunderstood, *“There was like a week of seeing everybody. And when they were coming back asking us do […] do you remember going into the POP trial and I'm thinking no, you know, I don't remember anything”* (Female 25). Three parents in the TIPIT trial told the researchers that they were comfortable with the limitations of their understanding because they felt that their baby was safe, *“I don’t understand a lot of the medical terms and things, but I know it’s not harming him or anything, so for me, you know, I was like, go ahead with it”* (Female 41). However, there were cases in all four trials where parents had specific misunderstandings, which had the potential to influence their decisions on trial entry (Table 3).

Perceived clinical benefit and access to medication were the most frequent parental misunderstandings. Regarding clinical benefit, parents, particularly from the TIPIT and MENDS trials, did not speak of the trial as having been designed to test whether or not the trial medication was beneficial to a group of children. Regarding access to medication, some parents in the MENDS trial were not aware that the trial drug was available outside the trial and only participated to access the drug, “*We’d already made our mind up that we were going to. Before we’d even got the information […] we just weren’t getting sleep […] it’s like, we have to do something”* (Female 22).

We labelled accounts of clinical benefit as misunderstandings only when parents (n = 8) emphasised a sense of certainty that their child stood to benefit from trial medication: “*if he does get the <Name of trial drug> on this trial it will help him have a good night’s sleep*” (Female 4). Other parents spoke of how they *hoped* their child would benefit (n = 7) and seemed to understand that there was a 50∶50 chance of getting the trial medication: “*I was just thinking I hope he gets the <Name of trial drug> one*” (Male 5). We did not label these accounts of hope for clinical benefit as misunderstandings because such hope is compatible with the rationale for conducting a clinical trial. In any event, such comments did not represent a direct misunderstanding of what parents had been told in the clinical trial discussions in the sense that practitioners often began trial discussions by mentioning previous research indicating the potential benefits of the trial medication.

Despite many practitioners clearly explaining how the randomisation process worked, some parents were confused. For example, some mistakenly believed that practitioners made the decision about which arm of the trial their child was allocated to, rather than allocation being conducted by computer randomisation: *“I wonder who does actually makes the decision, who goes on what and who doesn’t”* (Female 1). Finally, parents were concerned that trial procedures would be burdensome. Some parents only became aware of certain trial procedures during the course of the trial, whilst one parent declined the trial because she mistakenly believed that it involved extra invasive procedures (see Table 3). The trial medication was to be given through lines already in use to feed the baby, but this was insufficiently explicit in the trial discussion: *“It's called err a syringe pump basically, which is, you know, puts medicine to the veins”.* (Practitioner 2, trial discussion).

## Discussion

Our study has illuminated parents’ agendas when making a decision about participation in a paediatric clinical trial. Some of these agendas, including safety, trial purpose, practicalities of participation and randomisation, overlap with those conventionally prioritised by the research community [12], [17], [23], [36]. However, other agenda items, such as access to medication, clinical benefit, showing gratitude to a practitioner and practitioner opinion, differ from those prioritised by the research community. Our study provides additional evidence about what parents consider important when making a decision about a clinical trial [36]. However, perhaps its most significant contribution is in drawing attention to how parents’ agendas were often overlooked during interactions with practitioners. We also provide new evidence about how parents’ agendas were associated with specific misunderstandings, which in turn have the potential to influence parents’ decisions about a trial.

While some parental misunderstandings appeared to be linked to how practitioners had explained the trials, importantly, we also found that even when practitioners’ descriptions were clear, parents sometimes incorrectly interpreted the information provided. Moreover, our study showed how parents did not commonly seek clarification from practitioners or express their queries or concerns during the discussions that form a routine part of recruitment to most paediatric trials. Relational decision making was evident, as parents valued practitioner opinions, they trusted their judgement and made decisions without engaging with the finer details of the trial [37], [38]. Parents said little during the trial discussion and practitioners asked few open-ended questions, preventing the identification of misunderstandings [25]. Many of the parents’ questions and concerns only became apparent during the subsequent parent-researcher interviews.

Encouraging more parental participation in the discussions may help practitioners identify key issues and concerns for parents and provide appropriate information and clarification. Our study points to the potential benefits of practitioners tailoring information to what parents deem important as well as to the conventions of the research community [22], [36], [39]. However, as parents prioritised showing gratitude, practitioners may need to explain that parents should not feel obligated to participate because of the care their child received [40], [41]. In indicating that parents’ role in recruitment discussions was somewhat passive, our findings stand in contrast to previous literature which has characterised parents as taking an active role in recruitment discussions, and as valuing the ability of practitioners to listen to the questions and concerns of parents [42]. However, whereas previous research has tended to rely exclusively on parental reports of their experiences of recruitment, we also had access to recordings of trial recruitment discussions.

In line with previous literature, some parents were subject to the ‘therapeutic misconception’ as their decisions about trial entry were influenced by a belief that their child would benefit from trial medication [15], [19], [43]. However, we did not regard those who simply hoped that their child would benefit as having misunderstood the purpose of the trial – after all, no trial would be conducted unless there was reason to hope that it might offer clinical benefit. Parents understandably prioritised their child’s wellbeing and would only enter a trial if they felt their child would not be harmed; this included requiring reassurance that trial medication could be taken alongside other medication. Practical implications of involvement were important to parents, yet incorrect interpretations about trial procedures and protocol meant that parents declined unnecessarily or were not fully aware of what the study entailed [7], [10]. These types of misunderstandings have the potential to leave parents with negative views about research and impact upon trial enrolment and retention. Addressing these is a challenge for practitioners in conducting trials and evidence-based medicine in paediatrics as a whole.

Providing parents with sufficient time to digest written trial information and ensuring opportunities to ask further questions at a later date may further help to improve their understanding [15], [16]. However, parents may struggle to voice or formulate questions [44]–[47], which is understandable given that research will be a topic that is new to many. In our study practitioners asked parents few open-ended questions and parents initiated few questions, indicating the need to improve communication during trial discussions in order to identify and address parents’ misunderstandings [7], [25]. Others have suggested that this might include asking parents to describe their understanding of the trial in such a way that these are not perceived to be a ‘test’, such as: ‘I need to check that I have made everything clear enough. Could you tell me what you have understood?’ [10], [48] Alternatively, practitioners could simply ask parents open-ended questions and use prompts to invite opinions about the trial and explore understanding [10], [16], . Our findings suggest that such questions or prompts should cover a range of different issues, as parents in our study often had more than one agenda item. Practitioners might also assist parents by making simple adjustments to the way they invite questions (e.g. by ‘scaffolding’ their question invitations around particular aspects of the trial) and by linking their question invitations to the parental priorities identified in this study [51]. Author derived question examples, which could be used to assist communication during trial discussion are presented in Table 4. Question invitations could also be adapted to suit the individual trial and to help structure a recruitment interaction with a parent or child to assist communication and improve understanding [21], [22].

**Table 4:** Invitations to elicit parents’ questions in parent-practitioner discussions about clinical trials.

	Question
1	Based upon the information we have given you, what’s your opinion about this study?
2	I need to check that I have made everything clear enough. Could you tell me what is the purpose of this study?
3	Is there something you would like to ask me about the potential risks of the study?
4	Do you have some concerns about how taking part in the study may impact upon your child’s daily routine?
5	Do you have some questions about any medication that your child is currently taking and whether this needs to be reviewed if your child takes part in the study?
6	Do you foresee problems with administering the medication?
7	Have you had chance to talk to your husband/partner or a family member about the study?
8	Could you tell me whether you think your child may benefit from taking part in the trial?
9	Would you like me to go over again how children are placed into different groups to find out if this medicine/medical device is effective?
10	Is there anything you are worried about or would like me to go over again?

Further research is necessary to establish the effectiveness of these suggestions in assisting communication and parental understanding in paediatric clinical trial recruitment. Similarly, research is required to investigate alternative ways of presenting trial information outside of the recruitment discussion to address parental questions and concerns that may only become apparent to them after they have had time to reflect on the trial discussion, or when parents do not wish to directly ask a practitioner. Online formats such as web based forums and the use of social networking sites may also enable on going communication between parents and practitioners over the life of a trial [52], yet their feasibility and effectiveness is not known.

### Limitations

The study had some limitations. Mothers were over-represented in the sample as fathers were either not present in recorded trial discussions or did not participate in interviews. As a result, potential gender differences in the agendas and understanding of parents could not be explored. As gender has been shown to affect communication between patient and practitioners, with female patients receiving more information and asking more questions than males [53], [54], further research is needed to investigate fathers’ priorities and understanding of clinical trials. The impact of practitioner gender upon parent-practitioner communication and parental understanding should also be explored [55]. Although parents were accessed through multiple centres, the sample was weighted towards areas of higher material deprivation and participating hospitals were located in urban city centres. It was therefore not possible to fully explore potential differences in agenda items or levels of communication in parents from less deprived areas. Further research is required to fully explore links between material deprivation and geographical location and parental agenda items. While parents and practitioners may have several discussions about a trial, it was only possible to record one of these discussions for each family and this may have constrained our interpretations. In particular, it is possible that parents’ agendas were focussed upon issues during trial discussions that we did not audio record. However, relatively few studies of paediatric clinical trials have accessed trial discussions at all, with the result that these interactions have remained a “black box” thereby limiting the development of knowledge about how communication about clinical trials goes awry and how to enhance it [39]. Moreover, in recording both initial and subsequent discussions we have provided insights into parental agendas and misunderstandings at both time points. Quantification in social research has been subject to much debate [56], [57]. Our inclusion of frequencies (in Tables 2 and 3) for themes identified in qualitative analysis served to illustrate our interpretations and verify the basis for conclusions drawn [34]. However, these frequencies should be interpreted with caution. Whilst we incorporated four trials representing different conditions, disease status and designs to help maximise the transferability of findings we acknowledge that quantitative research is necessary to examine the wider generalizability of our conclusions. Data saturation was reached in terms of parent agendas. However despite our sample being relatively large for a qualitative study, our data on misunderstandings was more limited and saturation was not fully achieved. As other qualitative researchers have also described, resource constraints limited our ability to continue sampling in order to reach saturation for all categories [35]. We described variations and similarities in agenda items and misunderstandings within and between trials where appropriate, yet further research is required to explore agenda items and misunderstandings amongst parents’ recruited to different trials and settings (e.g. trials conducted in paediatric emergency care). Finally, our sample included few parents who had declined or withdrew or were ineligible for trials, insight into the agendas and misunderstandings amongst such groups of parents are needed to inform future recruitment practice.

### Conclusions

Our findings provide insights into parents’ agendas when making a decision about a clinical trial, evidence that their agendas often remain unvoiced in discussions with practitioners, as well as indicating the misunderstandings that can arise around parental agendas. These insights can be used by practitioners to structure trial recruitment discussions. Improved communication between practitioner and parent tailored to the needs and concerns of parents may improve recruitment practice and ultimately benefit evidence-based paediatric medicine.
